# Cystic Endometrioma with Coexisting Fibroma Originating in a Supernumerary Ovary in the Rectovaginal Pouch

**DOI:** 10.1155/2017/7239018

**Published:** 2017-01-22

**Authors:** Daiki Ogishima, Asumi Sakaguchi, Hiroko Kodama, Kanako Ogura, Ayako Miwa, Yayoi Sugimori, Shozo Matuoka, Toshiharu Matsumoto

**Affiliations:** ^1^Department of Obstetrics and Gynecology, Juntendo University Nerima Hospital, Tokyo, Japan; ^2^Department of Diagnostic Pathology, Juntendo University Nerima Hospital, Tokyo, Japan

## Abstract

A supernumerary ovary is an exceedingly rare disorder, in which the structure containing ovarian tissue is located at some distance from the normally placed ovary. 16 cases of endometriosis or tumors originating in a supernumerary ovary have been published in the English literature, but no case of coexisting endometriosis and a tumor has been published. We present the case of a 40-year-old female with cystic endometrioma with coexisting fibroma originating in a supernumerary ovary in the rectovaginal pouch. The present case is the first to be reported with coexisting endometriosis and a tumor originating in a supernumerary ovary. Our experience with this case and the results of our previous studies of rectovaginal endometriosis indicated that the possibility of originating in a supernumerary ovary shall be examined in cases of cystic endometrioma in the rectovaginal pouch.

## 1. Introduction

A supernumerary ovary is one of the rarest conditions of gynecological conditions, in which the structure containing ovarian tissue, such as the primordial follicle, is located at some distance from the normally placed ovary [1]. It occurs in the pelvic area, attached to the uterus, bladder, pelvic wall, retroperitoneum, and omental, mesenteric, and inguinal regions [2]. Tumors or endometriosis originating in the supernumerary ovary is exceedingly rare, with only 16 cases reported in the English literature [3–17]. Here, we present a case of cystic endometriosis with coexisting fibroma originating in a supernumerary ovary in the rectovaginal pouch and describe the differences between this case and rectovaginal endometriosis based on the results of our previous studies [18, 19].

## 2. Case Presentation

**Figure 1:**
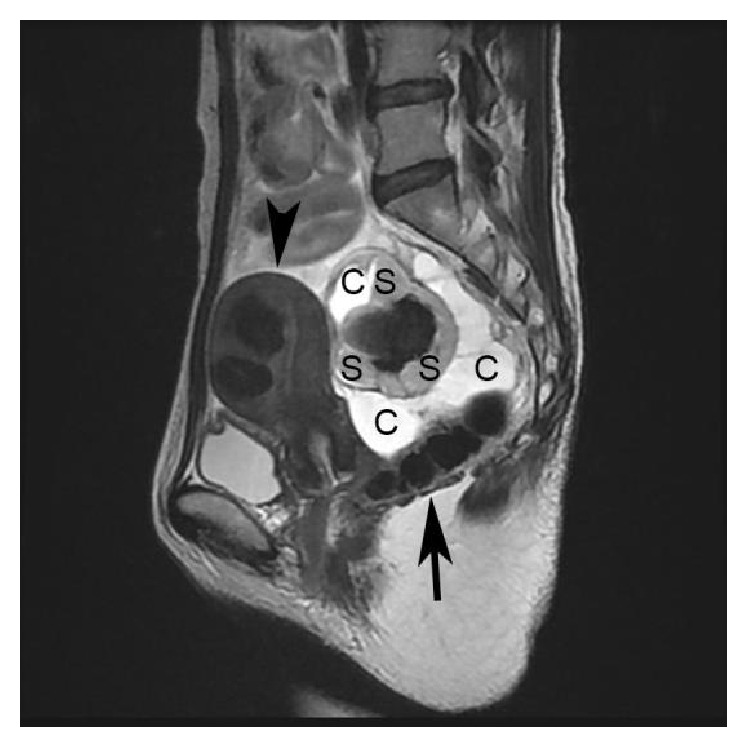
The rectovaginal mass on pelvic MRI (T2-weighted, sagittal line). The mass with multilocular cyst (indicated by C) and solid (indicated by S) components and a maximal diameter of 9 cm locates in the rectovaginal pouch. The uterus is indicated with an arrowhead, and the rectum is indicated with an arrow.

**Figure 2:**
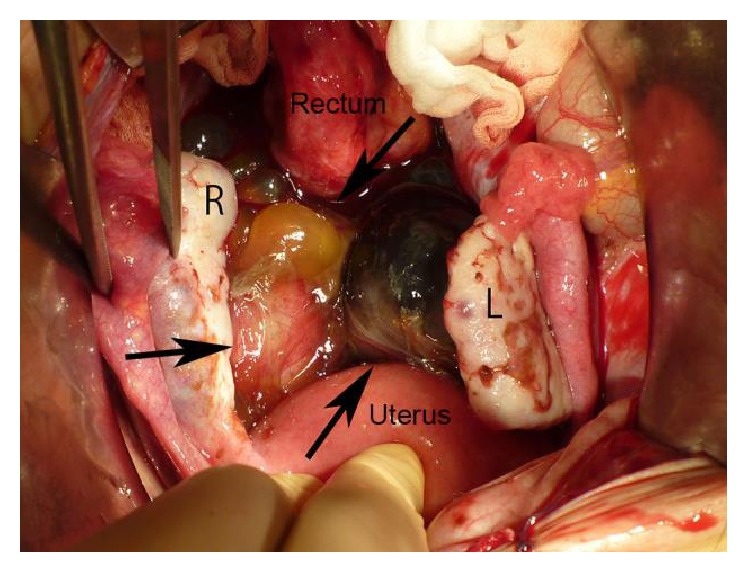
View of the rectovaginal pouch during the operation. The cystic and solid mass, indicated by arrows, is present in the rectovaginal pouch and it is located separately from normal bilateral ovaries (R, right ovary; L, left ovary).

A 40-year-old 2-multipara female was urgently admitted to Juntendo University Nerima Hospital with a diagnosis of acute abdomen. She had no previous pelvic surgery. Her initial laboratory data revealed a normal range of WBC and CRP and an elevation of CA125 (114 U/mL) and CA19-9 (402 U/mL). We had a diagnosis of the rupture or torsion of the ovarian malignant tumor because of the findings of magnetic resonance imaging revealing the suspicion of hemorrhage within the rectovaginal mass (Figure 1), and an operation was performed by the accommodation of the acute abdomen within one day after admission. During the operation, a small amount of yellowish ascites was pooled in the abdominal cavity. A cystic and solid mass, with a maximal diameter of 90 mm, was present in the rectovaginal region, the mass did not connect to the bilateral ovaries, and the bilateral ovaries and fallopian tubes showed no abnormality (Figure 2). The resection of the mass was performed by the separate resection of the cystic and solid portions, respectively. The resection was smoothly performed without adhesion to the surrounding peritoneum.

**Figure 3:**
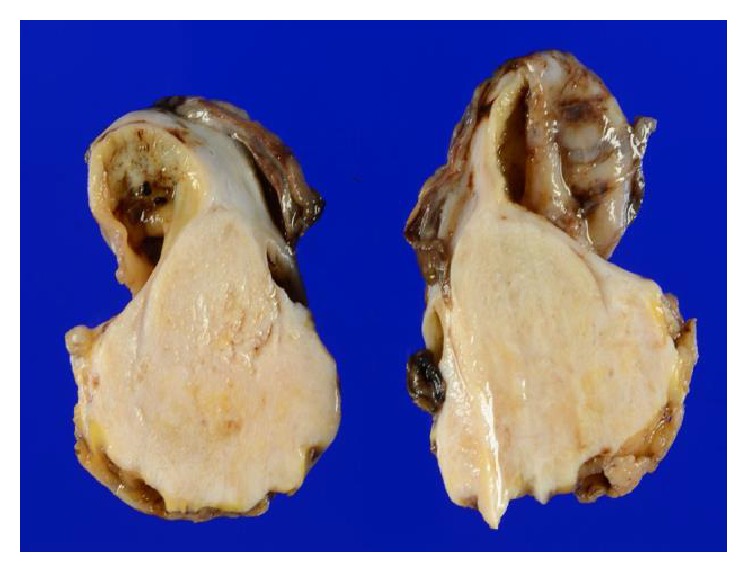
Gross appearance of the solid part of the rectovaginal mass in the cut section. Note the gray-colored fibrous appearance in the solid part.

After mass fixation by formalin solution, the size of the cystic portion was 35 × 30 mm and that of the solid portion was 50 × 45 × 30 mm. The solid area showed a gray-colored fibrous appearance (Figure 3). Histological examination was performed with the addition of immunostaining of CD10, ER, PgR, alpha-inhibin, desmin, and smooth muscle actin.

**Figure 4:**
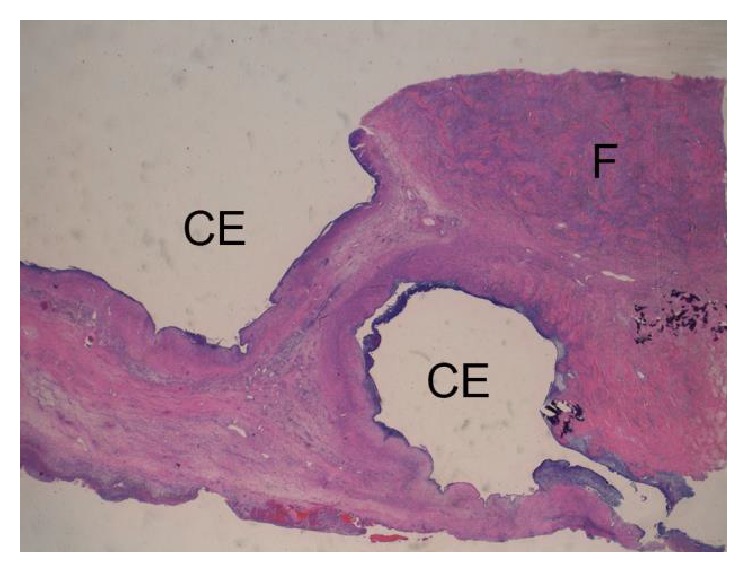
Loupe image of cystic endometriosis (indicated by CE) and fibroma (indicated by F). HE stain.

**Figure 5:**
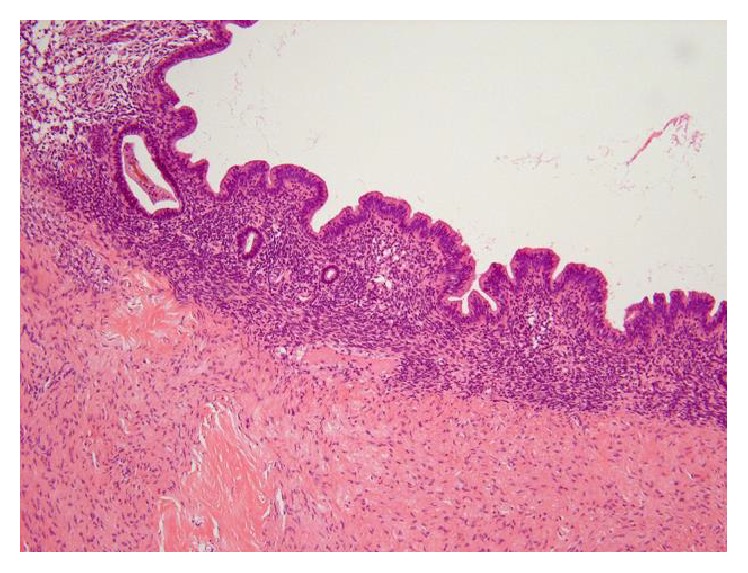
Endometrial tissue in the cyst wall of cystic endometriosis. HE stain.

**Figure 6:**
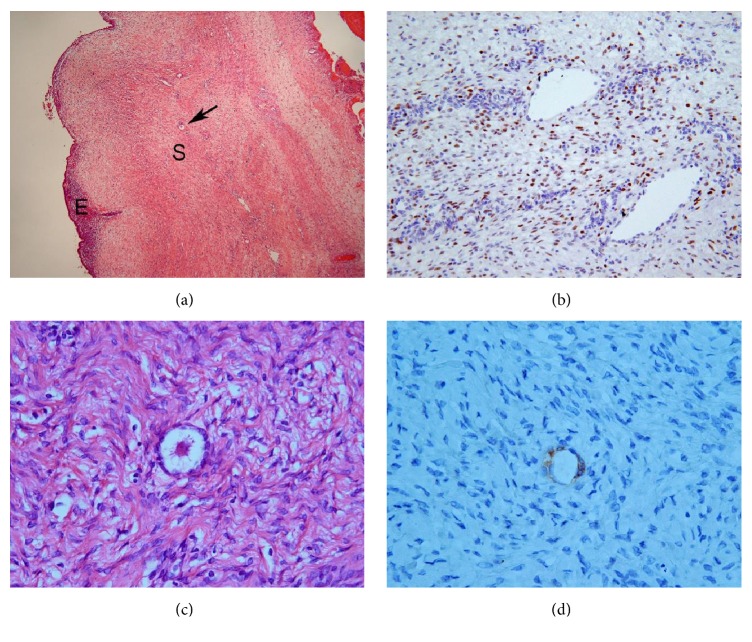
A primordial follicle in the ovarian stroma. (a) Note the spindle cell layer (indicated by S) around the endometrial tissue (indicated by E) of cystic endometriosis. A primordial follicle (indicated by an arrow) is present in the spindle cell layer. HE stain. (b) Many spindle cells show positivity for PgR immunostain, which indicates that the spindle cell layer is an ovarian stroma. (c) High-power view of primordial follicle. HE stain. (d) Granular cells consisting of primordial follicle show positivity for alpha-inhibin immunostain.

**Figure 7:**
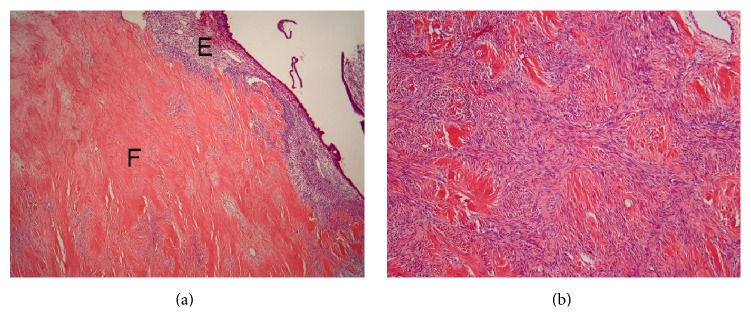
Histological appearance of the fibroma. (a) The fibroma (indicated by F) is present adjacent to endometrial tissue (indicated by E) of cystic endometriosis. HE stain. (b) High-power of the fibroma. Note the increase of the admixture of fibroblasts and collagen fibers. HE stain.

Histologically, in the cystic area, variously sized cysts with the lining of endometrial tissue in the cyst wall were present (Figures 4 and 5), which indicated a diagnosis of cystic endometriosis (endometriosis with cystic change). In the larger cysts, a spindle cell layer around the endometrial lining layer of the cyst wall was present (Figure 6(a)). The spindle cells showed positivity for PgR (Figure 6(b)) and week positivity for ER. In the spindle cell layer, the presence of the primordial follicle in a few sites was noted (Figures 6(a), 6(c), and 6(d)). Although the cellularity was lower compared with normal ovarian stroma, the spindle cell layer was considered as ovarian stroma because of the presence of primordial follicles and positivity for both PgR and EM in the spindle cells. In the solid area, spindle cells with bland nuclei and scant cytoplasm were arranged in intersecting bundles admixed with collagen (Figures 7(a) and 7(b)). The hyalinization of collagen bundles was noted at many sites, and calcification was focally present. These findings indicated that the solid part was fibroma.

Based on the clinical (solid and cystic mass in the rectovaginal region without connection to the normal bilateral ovaries) and histological (coexisting cystic endometrioma and fibroma) findings, the mass was diagnosed as cystic endometrioma with coexisting fibroma originating in a supernumerary ovary in the rectovaginal pouch.

This case was approved by the Research Ethics Committee of Juntendo University Nerima Hospital.

## 3. Discussion

Cases of lobulated, accessory, and supernumerary ovary are among the rarest conditions of abnormalities. A lobulated ovary is a normally situated ovary divided by one or several fissures into two or more lobes. An accessory ovary attaches to normal ovarian tissue or a ligament of eutopic ovary [2]. A supernumerary ovary is a similar structure but located at some distance from an eutopic ovary [2]; therefore this tumor was thought to be originated from supernumerary ovary.

The site and disease in the 17 cases (16 previously reported cases and the present case) with a tumor and/or endometriosis originating in a supernumerary ovary are presented in Table 1. Among the 16 previously reported cases, malignant tumor in 6 cases, benign tumor in 8 cases, and endometrioma in 2 cases occurred, respectively. Five of eight cases of the benign tumor were cystic teratoma, and fibroma, serous cystadenoma, and mucinous adenoma were a case each. (Table 1). Consequently, the present patient is the first reported case of endometrioma with coexisting fibroma originating in a supernumerary ovary.

In rectovaginal endometrioma, fibrosis occurred around the endometrial tissues and it extended into fat and connective tissue as well as within the endometrial tissues and finally produced a nodular fibrous mass, while causing clinical symptoms, including obstinate, severe dysmenorrhea, dyspareunia, and chronic pelvic pain [18, 19]. In the process of the disease progression of rectovaginal endometriosis, the dilation of endometrial glands occurred, but cyst formation, which is similar to endometrial cyst, did not occur [18, 19]. This cyst formation of endometriosis, that is, cystic endometrioma, observed in this case, is a characteristic finding, which is a point differing from rectovaginal endometriosis.

Concerning the diagnosis of this patient, we initially considered rectovaginal endometriosis with coexisting fibroma. However, cystic endometriosis in the rectovaginal pouch was not found in the 195 specimens of the 63 cases with rectovaginal endometriosis in our previous study [18]. So, we performed a detailed examination, including various histochemical stains, and found a few primordial follicles in the area of the cystic endometriosis, which led to the diagnosis of cystic endometriosis originating in a supernumerary ovary. These indicated the necessity of a detailed examination for the presence or absence of the ovarian tissue in cystic endometriosis in the rectovaginal pouch.

In rectovaginal endometriosis, fibrosis and smooth muscle metaplasia frequently occurred. So, concerning the pathogenesis of the coexisting fibroma in this case, metaplastic theory may be considered. However, fibroma is the most common ovarian stromal tumor [20], so the consideration of the coexisting fibroma arising from the ovarian stroma in a supernumerary ovary is more reasonable.

In conclusion, we reported the first case of cystic endometriosis with coexisting fibroma originating in a supernumerary ovary in the rectovaginal pouch. When cystic endometriosis is present in the rectovaginal pouch, our experiences with the present case suggest that the possibility of cystic endometriosis originating in a supernumerary ovary shall be examined.

## Figures and Tables

**Table 1:** Site and diseases in 17 cases with tumors and/or endometriosis originating in supernumerary ovary.

Case	Authors	Year	Age	Site	Diseases
1	Wharton [1]	1947	49	Pelvic	Granulosa cell carcinoma
2	Wharton [1]	1947	34	Side of the right ovary	Serous cystadenoma
3	Irving and Clement [2]	1967	21	Omentum	Cystic teratoma
4	Kriss [3]	1973	23	Omentum	Cystic teratoma
5	Hogan et al. [4]	1975	—	Omentum	Cystic teratoma
6	Printz et al. [5]	1977	48	Left retroperitoneum	Mucinous cystadenocarcinoma
7	Huhn [6]	1982	36	Left retroperitoneum	Mucinous cystadenoma
8	Roth and Ehrlich [7]	1987	34	Omentum	Cystic teratoma
9	Cruikshank and van Drie [8]	1991	—	—	Adenocarcinoma
10	Mercer et al. [9]	1992	47	Omentum	Cystic teratoma
11	El-Gohary et al. [10]	1995	32	Left retroperitoneum	Endometrioma
12	Barik et al. [11]	2001	47	Omentum	Fibroma, Meig's syndrome
13	Barik et al. [11]	2001	28	On pregnant uterus	Endometrioma
14	Besser and Posey [12]	2005	30	Left retroperitoneum	Mucinous adenocarcinoma
15	Badawy et al. [13]	2013	64	Retrouterine	Serous adenocarcinoma
16	Kamiyama et al. [14]	2013	31	Left retroperitoneum	Serous adenocarcinoma
17	Present case	2016	40	Rectovaginal pouch	Cystic endometrioma and fibroma
